# Integration of complementary and integrative medicine competencies in general practice postgraduate education – development of a novel competency catalogue in Germany

**DOI:** 10.1186/s12906-021-03419-7

**Published:** 2021-10-06

**Authors:** Jan Valentini, Carina Klocke, Corina Güthlin, Stefanie Joos

**Affiliations:** 1 Institute for General Practice and Interprofessional Care, University Hospital and Faculty of Medicine Tuebingen, Tuebingen, Germany; 2 Institute for General Practice, Goethe University, Frankfurt, Germany

**Keywords:** Integrative medicine, General practice, Family medicine residency, Postgraduate medical education, Medical education, competency catalogue

## Abstract

**Background:**

Complementary and integrative medical procedures (CIM) play an important role in general practice (GP). Consequently, in some countries (e.g. USA, Australia) specific curricula for the integration of CIM competencies in GP postgraduate education exist. Although Germany is one of the countries where CIM is strongly integrated in general practice, no such catalogue exists up to date. The aim of this study was to define a set of CIM competencies that are seen as relevant and feasible for postgraduate education in the German general practice setting.

**Methods:**

We used a multi-step, peer-based approach combining four different steps. Firstly, a survey among GP trainees (*n* = 138) was performed in order to assess needs and attitudes towards CIM. Then, existing competency-based CIM curricula were identified in international literature, translated into German and compared with the needs assessment from the survey. In a next step, we performed a survey among the CIM working group of the German Society for General Medicine and Family Medicine (DEGAM). As a last step, in a peer-based survey, GP trainers, GP trainees, and members of professional CIM associations (*n* = 131) evaluated a list of CIM competencies according to relevance and feasibility for general practice.

**Results:**

Within this multistage process, a final catalogue of 16 competencies was defined, covering the following areas: Medical knowledge, patient care and communication, practice-based learning, professionalism, and competencies based on the German healthcare system.

**Conclusion:**

The final catalogue of CIM competencies is intended to serve for GP training complementing the German competency-based curriculum for general practice. These competencies cover basic skills and are not intended to replace existing additional qualifications awarded by the medical associations in specific CIM methods, such as acupuncture or manual medicine. Therefore, a list of relevant competencies on CIM is available in order to serve as add-on for postgraduate education in general practice in Germany.

**Supplementary Information:**

The online version contains supplementary material available at 10.1186/s12906-021-03419-7.

## Introduction

International literature shows that complementary and integrative medical procedures (CIM) play an important role in general practice (GP) [1, 2]. When compared to other countries as the USA, the number of general practitioners (GPs) providing CIM in Germany is rather high. There is some evidence suggesting that up to 60% of GPs are using CIM in Germany [3–5]. In a systematic overview of nationwide surveys on the use of classical naturopathic treatments (NHV) and complementary and alternative therapies (CAM) by Linde et al., the proportion of the population that has used at least one of these methods varied between 40 and 62% [1]. In a systematic review for intra-European comparison, the use of CAM varied between 0.3 and 86% of the population. However, all authors emphasised the difficulty of estimating the utilisation because an umbrella term for CIM was lacking and, in connection with this, the studies were difficult to compare [6].

In order to deal with an increased demand on the one side and potential risks of CIM on the other side, some countries (e.g. USA, Australia to the best of our knowledge) have developed specific competency-based curricula for the integration of CIM competencies in GP postgraduate education over the last two decades.

For the first time in the USA in 2000, a list of 18 competencies in the field of complementary and alternative medicine for postgraduate education in family medicine was published, with the competencies each assigned to either attitudes, skills, and/or knowledge (ASK) [7]. In 2013 and on behalf of the Society of Teachers of Family Medicine (STFM), Locke et al. published a revised list of these competencies. When doing so, the overarching assignments to the ASK were removed and a total of 19 competencies were described. These competencies were assigned to different categories such as patient care, medical knowledge, interpersonal and communication skills, practice-based learning and development, professionalism, and systems-based care. In addition, a list of nine skills was described to support these 19 competencies [8]. Individual universities in the various American states were in charge of the implementation of this competency-based curriculum, which was exemplarily evaluated by Gardiner et al. in 2013. For this purpose, an online survey was conducted by the Council of Academic Family Medicine to elicit the knowledge and attitudes of residency faculty regarding CIM competencies as well as the inclusion of CIM in the local residency regulations. The results showed that the majority of the CIM competencies are known and considered to be an important part of the curriculum for future GPs. Various barriers (e.g. lack of time, lack of qualification of training officers, lack of access to CIM experts, etc.) which stood in the way of implementation were also identified [9]. A further development of a CIM curriculum was described by Lebensloh et al. in 2012: GP postgraduate trainees were able to complete parts of the curriculum via online training. The evaluation of this training demonstrated a positive assessment with regard to the feasibility of the online training, achievement of learning objectives, clinical benefit, and technical implementation by the GP postgraduate trainees [10]. In 2014, the same research group published data showing that the Integrative Medicine in Residency Curriculum is attractive to many medical students. It is also increasingly leading them to choose a residency in family medicine as the integrative medicine curriculum allows them to broaden their skills and knowledge in patient care as well as responds to the increasing interest in learning integrative medicine [11].

In Australia, the area of CIM was first included in the Curriculum for Australian General Practice for general medical education by the Royal Australian College of General Practitioners (RACGP) in 2007. Subsequently in 2016, the RACGP published a stand-alone chapter on integrative medicine [12]. Core competencies in general medicine for the area of CIM are described and adapted according to the respective level of medical training. These are acquired sequentially, built on each other, and completed throughout the entire medical education, postgraduate training, and continuing education. Thus, CIM has been successfully implemented throughout GP medical education in Australia [13].

In Germany, postgraduate training to become a GP is a structured and regulated five-year medical qualification with a final examination. The content of GP postgraduate education is regulated by the German Medical Association in the (Model) Specialty Training Regulations (MWBO) [14]. With the novel adaptation of the MWBO of 2018, Germany focusses on a competency-oriented approach [15, 16]. In addition, the German Society for General Medicine and Family Medicine (DEGAM) has developed a competency-based curriculum for General Medicine which defines, in addition to the MWBO, competencies for GP postgraduate education based on the CanMed roles, an outcomes-based framework of physician competencies [17–19]. After completing postgraduate education, physicians can take structured additional qualifications in the field of CIM, which are awarded by the medical association. The following additional qualifications in the field of CIM can currently be acquired in Germany after completion of a postgraduate training: Acupuncture, Homeopathy, Manual Medicine/Chirotherapy, Medical Balneology and Climatology, Naturopathic Methods, as well as Physical Therapy [14].

Up to date in Germany, there are some CIM topics integrated in undergraduate medical education in the German Medical Licensure Act. However, there is no such equivalent in GP postgraduate education (MWBO) in Germany. The competency-based curriculum for General Medicine of the DEGAM, includes a single competency labelled “complementary medical procedures” (chapter III.3) in the main chapter "therapy" without providing further details on its content [17]. More detailed information on CIM procedures or corresponding CIM competencies are neither included in the MWBO nor in the competency-based curriculum for GP postgraduate training of the DEGAM. Thus, a specific catalogue for teaching CIM competencies in GP postgraduate training is missing in Germany. Therefore, the aim of the present work is to close this gap and to develop a catalogue of CIM competencies for GP postgraduate education.

## Methods

### Multi-step, peer-based procedure

**Fig. 1:**
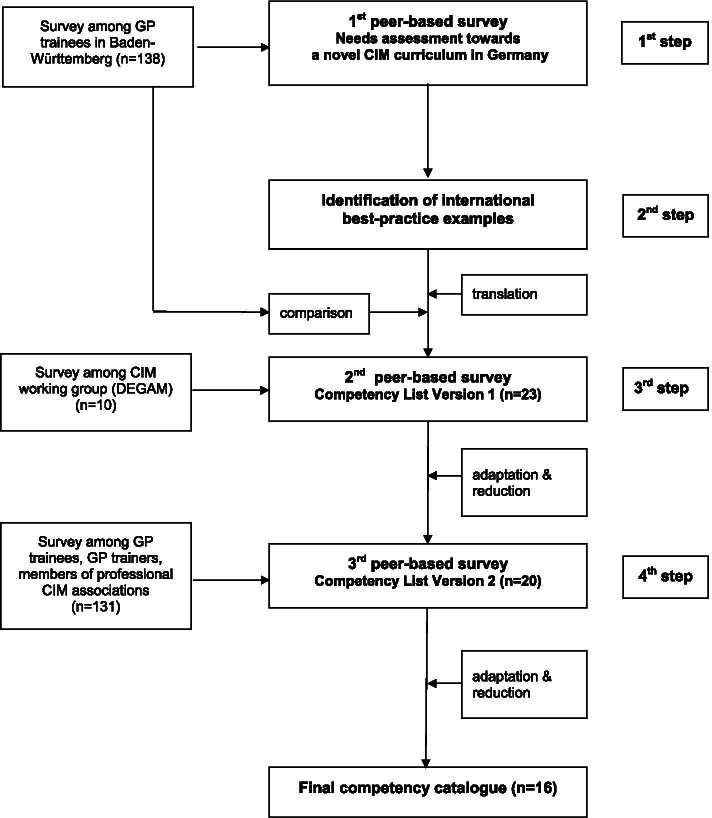
This figure illustrates the different action steps of the multi-step, peer-based procedure conducted to identify the competencies for the novel catalogue

In a multi-step, peer-based procedure, we addressed the question of which CIM competencies are seen as relevant and feasible for GP postgraduate education in Germany (see Fig. 1). Therefore, in a first step, a survey among GP postgraduate trainees in Baden-Württemberg, Germany (*n* = 138, overall response rate 28%), was performed in order to assess attitudes and needs towards CIM. The results showed a high interest in CIM as well as perceived uncertainty with regard to different practical topics. The vast majority of GP trainees would support the implementation of CIM training in the GP postgraduate training program. The results of this survey have been published previously by our study group in 2018 [20].

As a second step, existing CIM curricula were identified in a literature review. The competency-based curriculum of the STFM on integrative medicine was defined as a best practice example and used as a blueprint [8]. These competencies and learning objectives (19 basic competencies plus 12 supporting skills as, for example, *“Residents are expected to do the following: Demonstrate understanding of common complementary medicine therapies, including their history, theory, proposed mechanisms, safety/efficacy profile, contraindications, prevalence and patterns of use.”)* were translated into German. Then, these basic and supporting skills were compared with the needs assessment from the survey, combined in a single catalogue and adapted to the German context resulting in a list of 23 competencies.

This catalogue was then evaluated and further refined. As for the third step, a survey was performed within the Complementary Medicine Working Group of the German Society of General Practice (*n* = 10) [21]. The members were asked to rate the importance and feasibility of the included CIM competencies and were given the possibility to add free text comments or reword suggestions after each competency. Additionally, an overall rating of the importance of such a CIM catalogue for general medical education was to be given. The free text comments and ratings resulting from this survey were considered, and further adaption and reduction of CIM competencies yielded 20 competencies.

Finally, in our last step, an additional survey was performed in which the list of 20 competencies was evaluated with respect to relevance and feasibility for general practice by GP trainers, GP trainees and members of professional CIM associations. As this was the largest step in our multi-step, peer-based procedure and resulted in the novel CIM catalogue, we have described this in more detail below:

### Third peer-based survey

#### Participants and data collection

Participants of the third peer-based survey were GP postgraduate trainees enrolled in the postgraduate GP training program of the Competence Center for Postgraduate Medical Education in Baden-Württemberg, Germany (KWBW). The KWBW offers CIM workshops, amongst various topics related to family medicine [22]. All active GP postgraduate trainees (*n* = 432) of the KWBW and *n* = 52 graduates of the KWBW program received an email with an invitation to participate in the online survey. Furthermore, *n* = 250 GPs of teaching clinics of the University of Tübingen, enrolled in undergraduate and postgraduate medical education in Baden-Württemberg, Germany, received the invitation via email. Additionally, members of professional CIM associations from the Hufelandgesellschaft e.V., the largest German umbrella organization for integrative medicine societies, took part in the survey. The invitation to participate in the online survey was distributed through the newsletter of the Hufelandgesellschaft e.V. as well as through the newsletters of various affiliated CIM societies. The number of GPs who received the invitation via this latter channel cannot be determined.

#### Survey

The survey was launched in July 2019 and was accessible to participants until 31st January 2020. No email reminder was sent. Participation was voluntary, and the data were collected anonymously. In addition to ten sociodemographic questions, participants were asked to rate the importance and relevance for the daily work of 20 single CIM competencies on a four-point Likert-scale (1 = important, 4 = not important). The importance and relevance of the whole competency catalogue (i.e. the list of the 20 competencies as a whole) were also rated on the same four-point Likert-scale. Furthermore, the participants were asked two questions which could be answered with a free text, namely: *“Do you have further comments or suggestions for the novel CIM catalogue?”* and *“Is there anything missing?”*. Before implementation, the survey was given to two GP postgraduate trainees and to two GPs with no prior involvement on CIM education for pilot-testing with the think aloud method.

### Analysis and evaluation

Statistical analysis was conducted with SPSS Version 27 (SPSS INC.; IBM, USA). Mean, median, quartiles, absolute frequencies and percentages are reported according to the scale level. Subgroup analysis between GP postgraduate trainees and GP specialists was conducted with non-parametric Mann-Whitney U test. Subgroups smaller than *n* = 5 participants as well as cases with more than five missing values were excluded.

For the selection of the competencies for the final catalogue, the rate of consent (sum of participants rating an item as “important” or “rather important”, Likert-scale 1-2, in percent) was calculated for each item. A competency was selected for the novel catalogue if total consent was at least 80% or if total consent was < 80% but ≥80% among GP trainees in the subgroup analysis, as it reflects the needs of the target group. The overarching goal was to come up with the lowest number of competencies which are deemed to be relevant.

### Ethics

According to a communication from the Ethics Committee of the Medical University of Tübingen, according to the German federal law of § 3 Abs. 6 BDSG / LDSG BW, no formal ethical vote is required for the collection of anonymous data.

## Results

### Sociodemography

Overall, *n* = 131 participants joined the survey. The mean age of the participants was 49 years. Total 56% of participants identified as female, while within the group of GP trainees, the proportion of female participants was significantly higher with 73%. The proportion of participants within GP postgraduate education was 35%. More than one-third (38%) of the participants were GP trainers. The number of participants who hold one or more CIM specialization was almost 50%. As for CIM specializations awarded by the medical association, participants were mostly specialized in homeopathy (43%), acupuncture (40%) and naturopathy (34%). Almost 40% indicated other CIM related trainings, such as osteopathy and nutritional medicine (multiple responses possible). More than one-third (39%) of the participants were members of a professional CIM society. The vast majority of the participants was actively involved in patient care. About three quarters of the participants stated that they use CIM in a professional and personal context. Further details on the sociodemography are shown in Table 1.

**Table 1:** Sociodemographic characteristics

Characteristic	Participants (*n* = 131)^a^
Age, years (mean (SD))	48.9 (12.816), min. 27, max. 77
Sex, n (%)	
Diverse	1 (0.8%)
Female	72 (55.8%)
Male	56 (43.4%)
In postgraduate education, n (%)	
Yes	46 (35.1%)
	female 34 (73.9%) male 12 (26.1%)
No	85 (64.9%)
	diverse 1 (1.2%) female 38 (45.8%) male 44 (53.0%)
CIM Specialisation, n (%)^b^	
Yes	68 (51.9%)
	Acupuncture 27 (39.7%) Homeopathy 29 (42.7%) Manual Medicine/Chirotherapy 6 (8.8%) Medical Balneology and Climatology 2 (2.9%) Naturopathic Methods 23 (33.8%) Physical Therapy 1 (1.5%) Other, CIM related trainings 30 (44.1%)
No	62 (47.3%)
Patient care, n (%)	
Yes	123 (93.9%)
No	8 (6.1%)
Professional Use of CIM, n (%)	
yes	103 (78.6%)
No	28 (21.4%)
Private Use of CIM, n (%)	
yes	96 (73.3%)
No	35 (26.7%)
GP trainers, n (%)	
yes	48 (36.6%)
No	83 (63.4%)
CIM teaching, n (%)	
yes	26 (19.8%)
No	105 (80.2%)
Members of professional CIM associations, n (%)	
yes	51 (38.9%)
No	80 (61.1%)

^a^variable n due to missings (*n* = 130 to *n* = 131)

^b^multiple responses possible

### Evaluation of competencies

The ratings of every single competency are reported in Table 2. After applying the above described cut-off criteria, 16 out of 20 competencies were included in the final catalogue. These competencies are covering knowledge, skills and attitude (ASK) on the following CIM topics: Medical knowledge, patient care and communication, practice-based learning and continuing education, professionalism, and competencies with regard to the German healthcare system [23].

**Table 2:** Results of the evaluation of CIM competencies of the peer-based survey

GP trainees should…(competency)^a^	n	Mean (SD)	Quantile 50/25/75	(Rather) important %^**b**^
**1. Medical knowledge**				
*…be able to explain common CIM (complementary medicine, integrative medicine, naturopathy) concepts.* (q1)	131	1.74 (0.865)	2/1/2	**84.7**
*…be able to explain common CIM treatments including their respective theories, postulated modes of action, limitations.* (q2)	131	1.81 (0.842)	2/1/2	**83.2**
*…know the available evidence of effectiveness, interactions, and safety concerning the most common CIM concepts or understand where to find this information.* (q3)^a^	131	1.84 (0.812)	2/1/2	78.6
*…be able to give advice concerning CIM therapies for the most frequent consultation issues in family medicine.* (q4)	131	1.76 (0.802)	2/1/2	**83.2**
**2. Patient care and communication**				
*…conduct a biopsychosocial health interview, including aspects of lifestyle and usage of CIM.* (q5)	131	1.75 (0.788)	2/1/2	**84.7**
*…be able to jointly develop a treatment plan including conventional and complementary therapies with a patient and, if necessary, refer to appropriate facilities/therapists.* (q6)^a^	131	1.99 (0.907)	2/1/3	74.8
*…be able to inform patients critically about CIM treatments, which could potentially harm health and budget.* (q7)	131	1.41 (0.579)	1/1/2	**95.4**
*…be able to use non-pharmacological treatments (*e.g. *home remedies) for frequent issues of consultation (*e.g. *pain, fever, uncomplicated infections,* etc.*) or guide their patients thereto.* (q8)	131	1.21 (0.425)	1/1/1	**99.2**
*…be able to use common phytotherapeutics and supplements for frequent issues of consultation (*e.g. *pain, fever, uncomplicated infections,* etc.*).* (q9)	131	1.45 (0.623)	1/1/2	**94.7**
*…be able to consult regarding different relaxation techniques (meditation, mind and body practices, mindfulness, tai chi, yoga,* etc.*).* (q10)	131	1.88 (0.775)	2/1/2	**81.7**
*…be able to specifically apply placebo and self-efficacy effects as needed for the therapeutic process.* (q11)	131	1.60 (0.698)	2/1/2	**92.4**
**3. Practice-based learning**				
*…be able to use evidence-based sources of information concerning CIM.* (q12)	131	1.69 (0.692)	2/1/2	**88.5**
*…be able to identify their individual learning needs concerning CIM.* (q13)	131	1.89 (0.675)	2/1/2	**84.0**
**4. Professionalism**				
*…show respect and sympathy for patients‘interpretations of health, disease and suffering, based on individual attitudes and therapy requests concerning CIM.* (q14)	131	1.47 (0.636)	1/1/2	**93.9**
*…be open-minded and remain open to dialogue when it comes to another understanding of health and disease by medical and non-medical colleagues involved in a treatment.* (q15)	131	1.54 (0.694)	1/1/2	**90.1**
*…be able to take suitable action for self-care as needed.* (q16)	131	1.43 (0.621)	1/1/2	**94.7**
**5. Competencies based on the German healthcare system**				
*…know conditions and general framework of medical educations and medical specialist training (*e.g. *additional training) concerning CIM treatments.* (q17)^a^	131	2.37 (0.705)	2/2/3	57.3
*…know conditions and general framework of different professional groups offering CIM treatments (*e.g. *natural practitioners/Heilpraktiker).* (q18)	131	2.15 (0.815)	2/2/3	**67.9**^**c**^
*…know conditions and general framework for the medical practice concerning common CIM treatments (*e.g. *availability, prescription, legal regulations).* (q19)	131	1.90 (0.711)	2/1/2	**82.4**
*…consider conditions for patients’ access to complementary therapy care (*e.g. *remuneration and costs) during their treatment.* (q20)^a^	131	1.99 (0.775)	2/1/2	77.1

^a^Competencies in nonbold were not included into the novel CIM catalogue. Ratings in bold were included in the novel CIM catalogue

^b^Five-point Likert-scale was used: “(rather) important” reflects the rate of consent and is the sum of items “1 – important” and “2 – rather important”

^c^Competency was included after subgroup analysis; rate of consent among GP trainees 82.6%

The importance rating of the whole catalogue is high, 78% of all participants ranked it on the four-point Likert-scale as “1 = important” or “2 = rather important”. The relevance of the competencies for daily work presented in the survey was rated with 79% (important & rather important).

### Subgroup analysis of postgraduate physicians and specialists

A subgroup analysis of GP postgraduate trainees and GP specialists was performed. A significantly different rating was merely shown for questions no. 12 (“*GP trainees should be able to use evidence-based sources of information concerning CIM”*, U = 1283.000, Z = − 3567, *p* ≤ 0.05*)* and no. 18 (“*GP trainees should know conditions and general framework of different professional groups offering CIM treatments [e.g. natural practitioners/Heilpraktiker]”*, U = 1370.000, Z = − 3020, p ≤ 0.05*)*. The total consent as well as subgroup consents for question no. 12 was > 80%, it thus was selected for the novel catalogue. For question no. 18, total consent was < 80% (67.9%) for GP specialists but 82.6% within the group of GP postgraduate trainees. Therefore, it was included in the final list of competencies.

The final version of our competency-based catalogue on CIM in English language is presented in Table 3. The German version can be found in the supplementary files.

**Table 3:** Competency-based catalogue on complementary and integrative Medicine for GP trainees (English Version)

*GP trainees should…*	
Medical knowledge	
*1. …be able to explain common CIM (complementary medicine, integrative medicine, naturopathy) concepts.*	
*2. …be able to explain common CIM treatments including their respective theories, postulated modes of action, limitations.*	
*3. …be able to give advice concerning CIM therapies for the most frequent consultation issues in family medicine.*	
Patient care and communication	
*4. …conduct a biopsychosocial health interview, including aspects of lifestyle and usage of CIM.*	
*5. …be able to inform patients critically about CIM treatments, which could potentially harm health and budget.*	
*6. …be able to use non-pharmacological treatments (*e.g. *home remedies) for frequent issues of consultation (*e.g. *pain, fever, uncomplicated infections,* etc.*) or guide their patients thereto.*	
*7. …be able to use common phytotherapeutics and supplements for frequent issues of consultation (*e.g. *pain, fever, uncomplicated infections,* etc.*).*	
*8. …be able to consult regarding different relaxation techniques (meditation, mind and body practices, mindfulness, tai chi, yoga,* etc.*).*	
*9. …be able to specifically apply placebo and self-efficacy effects as needed for the therapeutic process.*	
Practice-based learning	
*10. …be able to use evidence-based sources of information concerning CIM.*	
*11. …be able to identify their individual learning needs concerning CIM.*	
Professionalism	
*12. …show respect and sympathy for patients‘ interpretations of health, disease and suffering, based on individual attitudes and therapy requests concerning CIM.*	
*13. …be open-minded and remain open to dialogue when it comes to another understanding of health and disease by medical and non-medical colleagues involved in a treatment.*	
*14. …be able to take suitable action for self-care as needed.*	
Competencies based on the German healthcare system	
*15. …know conditions and general framework of different professional groups offering CIM treatments (*e.g. *natural practitioners/ Heilpraktiker).*	
*16. …know conditions and general framework for the medical practice concerning common CIM treatments (*e.g. *availability, prescription, legal regulations).*	

## Discussion

In a rigorous multi-step, peer-based approach, a competency-based catalogue containing 16 basic competencies on CIM for GP postgraduate education in Germany has been developed. This approach was chosen analogously to the development of competency-based curricula successfully used in the field of general practice or spiritual care [24, 25].

Competency-based medical education has been firmly established in the Anglo-American world for some time and is internationally regarded as a showcase model [26]. In contrast, competency-based learning objectives have only recently been implemented for continuing education in Germany [15]. To the best of our knowledge, this is the first competency-based catalogue on CIM for GP postgraduate education in Europe. Within a multi-step, peer-based procedure, international best practice examples were translated and adapted to German context instead of reinventing the wheel. A similar approach was used by Steinhaeuser et al. for the development of the competency-based curriculum in general practice in Germany [24]. GP trainees and GP trainers were equally involved in the subsequent process of rating relevance and feasibility of potential competencies in order to get different perspectives. However, there are some limitations to this selection. Both groups may have had a special interest in CIM (also represented by the high percentage of participants with some form of CIM specialty), and thus may have overrated the relevance. However, to evaluate the specific competencies, a basic knowledge of CIM is essential. The participation in the survey was anonymous and voluntary and it was not possible to draw conclusions about individual participants. Therefore, the motivation of participants was unclear (“supporter or rejecter” of CIM). Additionally, the GP trainees and GP trainers were mainly located within the same federal state, where seminars on CIM topics had already been implemented in GP postgraduate training. Since several mailing lists were used for the recruitment of the participants of the third peer-based survey, the response rate cannot be determined.

For this third peer-based survey, we included GP trainees and GP trainers equally which showed a congruent rating for the majority of the competency assessments. One of the significant differences in the rating of competencies between these groups was competency no. 18, which related to competencies based on the German healthcare system. As GP trainees may not be familiar with all the conditions and the general framework of the German healthare system at the beginning of their professional career, they may have ranked the importance of these competencies higher. Similar uncertainties were congruently reported in a former needs assessment of GP trainees on CIM which was conducted by our study group [27]. As we focused on the ratings of GP trainees and GP specialists in order to cover the different levels of work experience, we did not distinguish gender differences during all stages of the multi-step, peer-based procedure.

As shown by Joos et al., CIM is already strongly integrated in primary care in Germany, although there is no specific CIM catalogue [3]. This discrepancy could also pose risks, especially since an evidence-based approach related to competencies for (the provision of) CIM was not part of their former GP training. In a qualitative study, Ostermaier et al. were able to show that almost all more experienced GPs integrated at least some CIM in their clinical practice with a high degree of pragmatism [28]. In contrast, young GP postgraduate trainees rate the importance of an evidence-based approach while providing CIM higher than their GP trainers. This could also be seen in our subgroup analysis on question no. 12 *GP trainees should be able to use evidence-based sources of information concerning CIM (q12)*, where a significant difference between the rating of GP trainees (97.8% rating (rather) important) vs. GP (83.5% rating (rather) important) could be shown. Similar results are also shown by a study group of Linde et al., where GP postgraduate trainees expressed doubts regarding the evidence-based approach of CIM and specific effects over placebo [29]. Consequently, GP postgraduate trainees may tend to be more critical towards CIM compared to their trainers or experienced GPs. Contrary to this finding, however, question no. 3 *GP trainees should know the available evidence of effectiveness, interactions, and safety concerning the most common CIM concepts or understand where to find this information,* was not rated with high importance and was therefore not included in the final catalogue, while competency no. 4 *GP trainees should be able to give advice concerning CIM therapies* […] (q4) was included. Another competency, which was not included in the final catalogue due to low rating, was question no. 6 …*GP trainees should be able to* […] *refer to appropriate facilities/therapists*. This could be explained by the fact that in Germany many GPs offer CIM themselves, and referrals to specific CIM practitioners are not common and therefore may not be considered relevant. Additionally, other professions that provide CIM in Germany, e.g. physiotherapists to some extent or natural practitioners (“Heilpraktiker”), do so in a setting outside of the statutory health care system and thus are directly accessible to the patient without a prior GP referral.

Furthermore, as an internationally accepted and commonly used definition of CIM is lacking, many procedures are considered part of this umbrella term and also brought up by patients during GP consultations; ranging from methods with strong evidence base, e.g. acupuncture and yoga, to methods where there are no or not enough data available to support their use, e.g. pranatherapy or ozone therapy. Therefore, addressing CIM in a neutral or even critical way and pointing to their limitations is an essential competency which was included in the final catalogue, e.g. by question nr. 2 *GP trainees should be able to explain common CIM treatments including their respective theories, postulated modes of action, limitations* and question nr. 5 *GP trainees should be able able to inform patients critically about CIM treatments, which could potentially harm health and budget*. Limitations and critical information are explicitly mentioned here.

Compared to the competency-based curriculum of the STFM on integrative Medicine, which was used as a blueprint for the present catalogue [8], we included a new competency in the category professionalism: no*.* 15 *GP trainees should be able to take suitable action for self-care as needed.* There is growing evidence showing that the work strain and burnout risk in GP postgraduate trainees, as well as in physicians in general, has been increasing over the last decades [30]. Therefore, promotion of physical and mental health seems to be essential in order to preserve the well-being of the physicians as well as their patients. This competency goes hand in hand with the principles of preserving health and well-being as described by Aaron Antonovsky in the basic concepts of Salutogenesis [31, 32]. These principles represent a fundamental approach in primary care and show a large overlap with many complementary medicine procedures (e.g. exercise, nutrition, and relaxation elements) underlining why CIM procedures are so prominent in GP. As further difference we proposed four competencies based on the German healthcare system in order to address the legal framework of CIM in Germany. Two out of these four competencies, covering regulatories concerning medical practice and knowledge upon other professional groups providing CIM methods, are listed in the final catalogue (competency no. 15–16).

The final competency-based catalogue on CIM is intended to complement the German competency-based curriculum for general practice of the DEGAM by serving as a kind of red thread for continuing education in the field of CIM or to be integrated into it [17]. The competencies included into the final catalogue are therefore rather meta learning objectives that do not substantiate which specific CIM method should be part of GP postgraduate education. To address this latter question, specific CIM approaches are covered by existing additional qualifications awarded by the medical associations (e.g. naturopathic methods) which we do not intend to replace.The potential addressees of such a novel CIM catalogue are GP postgraduate trainees, competency centers for postgraduate education in GP offering CIM teaching modules, as well as GP trainees and GP trainers providing continuing education in GP. The GP postgraduate training KWBW for example included CIM in their set of core competencies for becoming a GP [22]. However, as family medicine covers a wide range of medical fields, merely two teaching units of 45 minutes each could be budgeted for covering the core competencies in this field. Specific teaching modules need to be developed and implemented in order to make these CIM competencies achievable by GP trainees. Ideally, this could be accomplished by the use of appropriate entrustable professional activities combined with appropriate assessments [33, 34]. However, the conversion from a competency to specific teaching modules should be comprehensively elaborated, as the competencies implicate requirements, e.g. giving advice concerning CIM therapies (competency no. 4) presupposes the knowledge of the available evidence as a base.

Given the fact that structures in GP postgraduate training are already available, there are very good chances for the implementation of the catalogue. After implementation of this catalogue, further evaluation is needed in order to test the practicability of this catalogue. This may be done by assessing a gain of knowledge, attitude and/or skills in the area of CIM within GP trainees. This could ultimately lead to achieving the goal of improving patient care while addressing CIM topics. Finally, a full Kern-cycle, representing a six-step approach of curriculum development for medical education, could be completed [35].

## Conclusion

The competencies on CIM included in this novel catalogue are basic competencies intended to serve for GP training. They should be seen as general competencies for handling the growing demand on CIM in GP consultations with its diverse aspects; they are not intended to be learning targets relating to specific CIM methods. This catalogue of CIM competencies could be (partly) integrated in the existing German competency-based curriculum for general practice with the aim that they should be mastered by all GP postgraduate trainees by the end of their training. These basic competencies are not intended to replace the additional training in CIM awarded by the German medical associations e.g. of acupuncture or manual medicine. Additionally, some of the competencies may also be transferred to other medical specialties as e.g. internal medicine, psychosomatics or surgical specialties and therefore may be included into a variety of existing medical education programs. Altogether, this novel competency-based catalogue on CIM provides the foundation for a comprehensive and nation-wide competency-based GP postgraduate education on CIM in Germany.

## Supplementary Information

**Additional file 1: Supplementary Table 1.** Competency-based catalogue on complementary and integrative Medicine for GP trainees (German Version - Kompetenzbasiertes Katalog zur Komplementären und Integrativen Medizin für Ärztinnen und Ärzte in Weiterbildung zum Facharzt für Allgemeinmedizin).

## Data Availability

The novel Competency-based catalogue on complementary and integrative Medicine for GP trainees can be downloaded in English and German language here: https://www.medizin.uni-tuebingen.de/de/das-klinikum/einrichtungen/institute/allgemeinmedizin/forschung/komplementaere-und-integrative-medizin/kompetenzbasiertes-curriculum-zur-komplementaeren-und-integrativen-medizin The datasets and further additional files used and/or analysed during the current study are available from the corresponding author on reasonable request.

## Abbreviations

ASK: Attitudes, skills, and/or knowledge
BDSG: Federal Data Protection Act [*Germa**n*: Bundesdatenschutzgesetz].
CAM: Complementary and alternative therapies
CIM: Complementary and integrative Medicine
DEGAM: German Society for General Medicine and Family Medicine [*German*: Deutsche Gesellschaft für Allgemeinmedizin und Familienmedizin]
GP: General practice
GPs: General practitioners
KWBW: Competence Center for Postgraduate Medical Education in Baden-Württemberg, Germany [*German*: Kompetenzzentrum Weiterbildung Baden-Württemberg]
LDSG BW: State Data Protection Law Baden-Württemberg [*German*: Landesdatenschutzgesetz Baden-Württemberg]
MWBO: (Model) Specialty Training Regulations [*German*: (Muster-)Weiterbildungsordnung]
RACGP: Royal Australian College of General Practitioners
SD: Standard deviation
STFM: Society of Teachers of Family Medicine
