# Supplementary material for: Integration of complementary and integrative medicine competencies in general practice postgraduate education – development of a novel competency catalogue in Germany
Source: BMC Complement Med Ther. 2021 Oct 6;21:250. doi: 10.1186/s12906-021-03419-7 (PMC8496071; doi:10.1186/s12906-021-03419-7)
Supplement: Supplementary file 1 — Additional file 1: Supplementary Table 1. Competency-based catalogue on complementary and integrative Medicine for GP trainees (German Version - Kompetenzbasiertes Katalog zur Komplementären und Integrativen Medizin für Ärztinnen und Ärzte in Weiterbildung zum Facharzt für Allgemeinmedizin). [file 12906_2021_3419_MOESM1_ESM.docx]

## Additional file 1:

Supplementary table 1: Competency-based Catalogue on Complementary and Integrative Medicine for GP trainees (German Version *- Kompetenzbasiertes Katalog zur Komplementären und Integrativen Medizin für Ärztinnen und Ärzte in Weiterbildung zum Facharzt für Allgemeinmedizin*)

| *Ärztinnen und Ärzte in Weiterbildung Allgemeinmedizin sollen…* |
| --- |
| Allgemeines Wissen zu KIM |
| 1. *…die gängigsten Begrifflichkeiten in Bezug auf KIM (z.B. Komplementäre Medizin, Integrative Medizin, klassische Naturheilverfahren) erklären können.* |
| 1. *…die gängigsten komplementärmedizinischen Verfahren, einschließlich ihrer Theorie, postulierten Wirkmechanismen und Grenzen erklären können.* |
| 1. *…bei den häufigsten Beratungsanlässen in der Allgemeinmedizin zu gängigen komplementär-medizinischen Therapien beraten können.* |
| Patientenversorgung und Kommunikation |
| 1. *…eine biopsychosoziale Anamnese durchführen, die auch Angaben zum Lebensstil sowie der Inanspruchnahme von komplementärer Medizin umfasst.* |
| 1. *…Patientinnen und Patienten über Angebote von KIM, die zu möglichen gesundheitlichen und finanziellen Schäden führen können, kritisch aufklären können.* |
| 1. *…nicht-medikamentöse Behandlungen (z.B. Hausmittel) bei häufigen Beratungsanlässen (z.B. Schmerzen, Fieber, unkomplizierte Infekte etc.) anwenden bzw. Patientinnen und Patienten anleiten können.* |
| 1. *…gebräuchliche Phytotherapeutika und Nahrungsergänzungsmittel bei häufigen Beratungsanlässen (z.B. Schmerzen, Fieber, unkomplizierte Infekte etc.) anwenden können.* |
| 1. *…verschiedene Entspannungstechniken (z.B. Meditation, Mind-Body-Techniken, Achtsamkeit, Tai Ji, Yoga etc.) beraten können.* |
| 1. *…Effekte von Placebo und Selbstwirksamkeit bei Bedarf für den Therapieverlauf gezielt einsetzen können.* |
| Praxisbasiertes Lernen und Weiterbildung |
| 1. *…Evidenzbasierte Informationsquellen für den Bereich KIM nutzen können.* |
| 1. *…den persönlichen Lernbedarf im Zusammenhang mit komplementärmedizinischen Verfahren identifizieren und ausgleichen können.* |
| Professionalität |
| 1. *…Respekt und Verständnis für die Interpretationen von Gesundheit, Krankheit und Leiden der Patientinnen und Patienten zeigen, die auf individuellen Überzeugungen und Therapiewünschen bezüglich KIM beruhen.* |
| 1. *… Offenheit und Dialogbereitschaft gegenüber ärztlichen und nicht-ärztlichen Mitbehandelnden zeigen, die ein anderes Verständnis von Gesundheit und Krankheit haben als man selbst.* |
| 1. *… Geeignete Maßnahmen zur Selbstfürsorge bei Bedarf anwenden können.* |
| Kompetenzen bezüglich des deutschen Gesundheitssystems |
| 1. *…Regelungen und Rahmenbedingungen von verschiedenen Berufsgruppen kennen, die Methoden aus dem KIM-Spektrum anbieten (z.B. Heilpraktikerinnen und Heilpraktiker).* |
| 1. *…Rahmenbedingungen für die ärztliche Vorgehensweise im Hinblick auf die gängigsten KIM Verfahren (z.B. Verfügbarkeit, Verschreibung, gesetzliche Bestimmungen) reflektieren.* |
